# Impact of ethanol as a vehicle for water-insoluble pollutants in BEAS-2B cell toxicity assays

**DOI:** 10.1080/15376516.2025.2540457

**Published:** 2025-08-04

**Authors:** Emma Ann Landskroner, Candace Su-Jung Tsai

**Affiliations:** Department of Environmental Health Sciences, Fielding School of Public Health, University of California, Los Angeles, Los Angeles, CA, USA

**Keywords:** Ethanol, dissolution agent, *in vitro*, cytotoxicity, BEAS-2B, cosolvent

## Abstract

*In vitro* human cell models are the gold standard for toxicological screening of environmental pollutants, allowing precise profiling of cellular responses. Pollutants with limited water solubility require carrier vehicles for uniform exposure. Ethanol, a commonly used vehicle, is typically maintained at 0.05–1.0% (v/v) to minimize toxicity. However, definitive no-observed-adverse-effect levels (NOAELs) or lowest-observed-adverse-effect levels (LOAELs) for ethanol in non-tumorigenic human bronchial epithelial (BEAS-2B) cells, prevalent in inhalation studies, have not been established. Researchers thus apply a range of ethanol concentrations derived from diverse cell lines, increasing the risk of vehicle interference. This study evaluated ethanol as a cosolvent vehicle for four emerging high-flashpoint hydrocarbon (HFHC) dry cleaning solvents in BEAS-2B cells. HFHC solvents were solubilized 1:1 in 100% ethanol, then diluted in bronchial epithelial cell growth basal medium to final concentrations of 0.05%, 0.25%, 0.5%, and 2.5% (v/v). Vehicle, positive, and negative controls isolated ethanol-specific cytotoxic effects. Cytotoxicity was assessed via cellular viability (MTS assay) at 24 and 48 h, and lactate dehydrogenase (LDH) and interleukin-8 (IL-8) release after 24 h. Ethanol drove viability loss at ≥0.5% (24 h) and ≥0.25% (48 h), induced inflammation at concentrations ≥0.05%, and minimally impacted membrane integrity. Most HFHC solvents showed minimal effects beyond ethanol alone, except one HFHC, Intense, causing significant membrane disruption and cytotoxicity even at low doses (0.05–0.25%). Practical ethanol noninterference thresholds recommended are ≤0.5% for 24-hour assays, ≤0.25% for 48-hour viability, and ≤0.05% for inflammatory endpoints, establishing critical guidelines for ethanol use in BEAS-2B assays.

## 1. Introduction

Human cell cultures have long been employed as predictive models for assessing the toxicological and biological impacts of pollutant exposure on human health. In these *in vitro* studies, solvents such as ethanol, dimethylsulfoxide (DMSO), methanol, or polyethylene glycol (PEG) are standardly used as cosolvents to bridge polar and nonpolar phases; these water-miscible carriers enable delivery of lipophilic compounds into aqueous bioassay environments (Rais et al. 2008; Timm et al. 2013; Hamzeloo-Moghadam et al. 2014; Jamalzadeh et al. 2016; Toulouse et al. 2017; Modrzyński et al. 2019). Among carrier options, ethanol stands out – in a comparison study of seven vehicle solvents, it consistently performed the best across three of the four cell lines (HaCaT, A-375, A-431), with minimal impact on cell proliferation at up to 2.0% (v/v) (Ilieva et al. 2021). As a polar protic solvent, ethanol acts as an amphiphilic agent – its hydrophilic hydroxyl and hydrophobic ethyl group disrupt intermolecular forces in poorly soluble compounds, facilitating even dispersion (Miyako et al. 2010; Bagchi 2013; Green and Wheeler 2013; Hyde et al. 2019; Hansen et al. 2022). This results in the formation of a homogeneous stock solution that can then be further diluted with culture medium to achieve the desired working concentrations.

Universal guidelines for employing any dissolution agent in *in vitro* exposure studies are to maintain vehicle concentrations at the lowest feasible levels to prevent confounding cytotoxicity. Studies have sought to define the highest permissible ethanol concentration for the dissolution of water-insoluble compounds while remaining nontoxic, but reported thresholds vary depending on the cell type, tissue of origin, and experimental conditions (Wang et al. 2011; Timm et al. 2013; Nguyen et al. 2020; Ilieva et al. 2021; Koc et al. 2022). Ethanol vehicles tend to be used between 0.05% and 1.0% (v/v), although outliers report no cytotoxicity at levels as high as 2.0% or 2.8% (v/v) (Jain and Pento 1991; Wakabayashi and Negoro 2002; Wu et al. 2011; Timm et al. 2013; Kar et al. 2021). Still, the use of ethanol near the upper end of this range remains a concern, as it is shown to interfere with cellular signaling, barrier function, or cytokine release, even when cell viability appears unaffected (Ma et al. 1999; Jonsson and Palmblad 2001; Chopyk et al. 2017).

With improved physiological relevance and predictive accuracy, non-tumorigenic human cell lines have become a benchmark for *in vitro* toxicological screening (Grafström 1990; Rhim 1993; Zavala et al. 2020; Li et al. 2024). Unlike tumorigenic or animal-derived cells, these models more closely mimic the biological response of normal human tissues (Pamies and Hartung 2017; Madorran et al. 2020). Non-tumorigenic cells maintain greater genome stability across passages, preserve signaling pathways, and retain human-relevant metabolic capabilities, making them valuable for mechanistic toxicology and environmental health research (Nelson et al. 2017; Madorran et al. 2020; Trisolini et al. 2025). In particular, human bronchial epithelial cell line (BEAS-2B), an established non-tumorigenic type derived from healthy respiratory tract tissue, has been extensively applied to investigate inhalation toxicants (Garcia-Canton et al. 2013; Grilli et al. 2018; Han et al. 2020). The use of BEAS-2B has yielded vital insights into pollutant mechanisms of action, including NF-kB-mediated IL-6/IL8 release upon PM_2.5_ exposure, chromate-induced gene expression shifts, and epidermal growth factor receptors (EGFRs)-dependent migratory changes following chronic arsenic exposure (Sun et al. 2011; Wang et al. 2019; Kim et al. 2021). Transcriptomic analyses have further shown that BEAS-2B cells most closely resemble primary human bronchial epithelial cells, displaying significantly fewer dysregulated genes than tumor lines, genome integrity, and the ability to express functional metabolizing enzymes that stimulate biotransformation processes (Zimonjic et al. 2001; Ertel et al. 2006; Courcot et al. 2012; Agus et al. 2013; Coppola et al. 2022). Their application in toxicological studies has deepened our understanding of lung cell physiology and structure, uncovering cellular responses that signal the early stages of respiratory disease and lung carcinogenesis following toxicant inhalation (Garcia-Canton et al. 2013; Park et al. 2015; Han et al. 2020).

Limited studies have evaluated the cytotoxicity of ethanol as a cosolvent on BEAS-2B cell lines. Of those, ethanol at 0.1% (v/v) is primarily non-cytotoxic, showing no effect on viability, morphology, or cytokine release (Ertel et al. 2006; Sun et al. 2011; Lau et al. 2012; McCarrick et al. 2024). Concentrations between 0.2% and 0.5% (v/v) also preserve viability but disturb tight junction integrity and transepithelial resistance, indicating disrupted barrier function without overt cytotoxicity (Simet et al. 2012). Other studies have found that ethanol at 0.5–0.7% (v/v) suppresses IL-6, IL-8, and TNF-α responses via cAMP/PKA signaling, again without cell death (Wyatt et al. 2017). At 1.0% (v/v), ethanol has been tolerated in 24–48 h exposures, and one study reported no change in impedance even at 2.0% (v/v) (Wyatt et al. 2017; Zinkl 2022; Uski et al. 2024). Previous findings suggest that even non-lethal ethanol doses can alter cellular function, highlighting the need for endpoint-specific validation in BEAS-2B models.

Currently, no studies have definitively identified a no-observed-adverse-effect level (NOAEL) or a lowest-observed-adverse-effect level (LOAEL) for ethanol cosolvent in BEAS-2B cells. This absence of a well-defined threshold for noninterference has led to inconsistent concentration use and the application of analogous limits from other cell types, risking variability. Without a baseline understanding of BEAS-2B-specific thresholds, the likelihood of vehicle-driven interference increases, potentially compromising assay accuracy and impeding the ability to isolate authentic cytotoxic responses induced by water-insoluble pollutants.

### 1.1. Purpose and scope

This study examines the role of ethanol as a vehicle for solubilizing water-insoluble high-flashpoint hydrocarbon (HFHC) solvents in BEAS-2B cells, focusing on how different ethanol concentrations impact cellular viability, cellular membrane integrity, and pro-inflammatory signaling. The aim is to distinguish the effects induced by ethanol from those of HFHC test compounds, establish a NOAEL and LOAEL to ensure accurate toxicological assessment of these emerging dry-cleaning chemicals and other hydrophobic pollutants that will necessitate *in vitro* BEAS-2B assessment in the future.

## 2. Materials and methods

### 2.1. Cell line culturing

BEAS-2B cells (ATCC CRL-3588) were cultured in sterile, untreated T25 flasks utilizing bronchial epithelial cell basal medium (BEBM) supplemented with the Bronchial Epithelial Growth Kit (Gibco, Thermo Fisher Scientific, Waltham, MA). To prepare the flask culturing surface, a coating bed solution composed of the complete medium, fibronectin human protein, collagen I – rat tail, and AlbuMAX^™^ lipid-rich bovine serum was added to the uncoated T25 flask and distributed evenly to create a conducive coating bed for cell attachment (Gibco, Thermo Fisher Scientific, Waltham, MA). Cells were incubated at 37 °C in an atmosphere containing 5.0% CO_2_ and monitored daily, with medium changes occurring every two to three days depending on cell density and medium conditions. Upon reaching approximately 80% confluency, the cells were detached using a trypsin–EDTA (0.25%) solution and subcultured into a sterile T75 flask with an added coating bed solution to maintain growth and viability. Cells were counted to determine their initial seeding density to inform the calculation of the density needed for each exposure period to ensure uniformity in the final cell numbers across the different treatment groups. Cells were then seeded into 96-well plates at a density of 10,000 cells per well and incubated for 24 h to allow for reattachment in complete culture medium.

### 2.2. Solvents and preparation

HFHC solvents are up-and-coming aliphatic petroleum-based hydrocarbons advertised as nontoxic and less volatile than other dry-cleaning substances; however, their toxicological profiles remain largely uncharacterized (Toxics Use Reduction Institute (TURI) 2012; Ceballos et al. 2016; Whittaker 2019). Four emerging trade name HFHC solvents (DF-2000^™^, EcoSolv^®^, Calypsolv^™^, and Intense^®^) used in the laundry and dry-cleaning industry were donated for examination from facilities in the greater Los Angeles area. These solvents represent water-insoluble pollutants, for which toxicological data are lacking, necessitating the use of assisted dissolution. Ethanol 100% (Lab Alley, Austin, TX) was obtained as the cosolvent agent. Preliminary solubility testing confirmed complete miscibility of each HFHC solvent in ethanol at a 1:1 (v/v) ratio, chosen based on literature precedent and observed phase stability upon subsequent undisrupted dilution in BEBM. Each stock solution was prepared by mixing equal volumes (10 mL each) of HFHC solvent and ethanol. These stocks were then diluted into BEBM to yield final stock concentrations of 0.1%, 0.5%, 1.0%, and 5.0% (v/v) per well, resulting in corresponding ethanol and HFHC exposure of 0.05–2.5% (v/v) each. Matched vehicle controls (VCs) consisted of ethanol only at corresponding concentrations to isolate cosolvent-specific effects. Zinc oxide (ZnO) nanoparticles were used as positive controls (PCs), an established cytotoxicant to BEAS-2B, selected for their frequent use as a reference standard in cytotoxicity and genotoxicity studies involving this line (Xia et al. 2008; Heng et al. 2010, 2011). Negative controls (NCs) were also implemented, provided solely with BEBM medium.

### 2.3. Exposure and evaluation

BEAS-2B cells were exposed to ethanol-HFHC mixtures at final well concentrations of 0.05%, 0.25%, 0.5%, and 2.5% (v/v) for each component. Ethanol-only VCs were included at each matching concentration to identify potential confounding effects from the cosolvent. Cytotoxicity was evaluated using two 3-(4,5-dimethylthiazol-2-yl)-5-(3-carboxymethoxyphenyl)-2-(4-sulfophenyl)-2H-tetrazolium (MTS) Assay Kits (ab197010; Abcam, Cambridge, UK), performed at 24 and 48 h to capture initial and prolonged effects on metabolic activity. The lactate dehydrogenase (LDH) Colorimetric Assay Kit (ab102526; Abcam, Cambridge, UK) was used to measure real-time apoptosis and necrosis of the cell membrane over approximately one hour following 24 h of exposure. To assess inflammatory oxidant response, interleukin-8 (IL-8) chemokine secretion was measured using the Human IL-8 ELISA Kit (KHC0081; Thermo Fisher Scientific, Inc., Waltham, MA) at 24 h post-exposure. These assays were chosen to collectively assess cellular viability, membrane damage, and pro-inflammatory signaling, all being markers of toxicological disruption. Each reading was obtained using a Molecular Device SpectraMaxM5^e^ plate reader, set according to each test manufacturer’s specifications.

### 2.4. Statistical analysis

Each experiment included at least three replicates per concentration, except for the 48-hour MTS data, which had two replicates. Statistical analyses were performed using Python (version 3.9). Independent *t*-tests and one-way ANOVA were conducted in SciPy.stats, while two-way ANOVA with type II sums of squares was performed in Statsmodels to evaluate the main effects of solvent type and concentration, and their interaction on cell viability, membrane integrity, and inflammatory response. Post hoc pairwise comparisons were conducted using *t*-tests with Bonferroni’s correction to identify significant differences between conditions. Concentration–effect analyses within the VC group and correlational analyses between HFHCs and the VC responses were also performed. Statistical significance was uniformly set at *p* < 0.05. Descriptive results are expressed as the mean ± standard error.

## 3. Results

### 3.1. MTS assay: 24-hour and 48-hour exposure

After 24 h of exposure, the ethanol VC at 0.05% (v/v) caused no loss in viability (100 ± 0.0%). The hydrophobic HFHC solvent treatments at the same low dose (each delivered in ethanol) similarly showed high cell viability, with three of the four (DF-2000, EcoSolv, and Calypsolv) averaging ~84% viable cells. Intense, however, was a notable outlier even at 0.05%, reducing viability to 60.3 ± 8.2%. This resulted in an overall HFHC group mean of 78.2 ± 5.3%, slightly lower than the ethanol-alone VC (Welch’s *t*-test *p* ≈ 0.04, which did not remain significant after Bonferroni’s correction).

As the ethanol concentration increased, cell viability declined in a dose-dependent manner across both the VC and HFHC-exposed cells. At 0.25%, VC viability remained high (93.4 ± 3.7%) while the HFHC treatments averaged 75.7 ± 9.4%. By 0.5%, VC viability was 88.8 ± 8.3% versus a HFHC mean of 70.4 ± 5.2%. At the highest concentration (2.5%), viability dropped sharply to 59.5 ± 1.3% in the VC, with a comparable average of 57.7 ± 1.3% across the HFHC conditions, indicating extensive cytotoxicity regardless of the solvent present. In the 48-hour exposure duration group, cells were further sensitized. Even a 0.05% ethanol exposure reduced viability (VC: 89.8 ± 11.0% vs. HFHC group mean ~73.3 ± 22.0%, the latter lowered by Intense’s continued toxicity). At the highest dose (2.5%), the VC fell to 17.1 ± 3.4%, and the HFHC-treated cells similarly presented near-total cell loss, with a mean viability of 15.5 ± 6.0%. This confirms profound cytotoxicity with prolonged high-dose exposure. Tables 1 and 2 summarize cell viability after 24 and 48 h, expressed as mean cell viability (%) ± standard error.

Statistical analysis confirmed that ethanol concentration was the primary driver of viability loss, with no significant differences attributable to the HFHCs themselves. Two-way ANOVA identified highly weighty effects of both solvent type and concentration on viability at 24 h (solvent: *F*(4,40) = 15.68; concentration: *F*(3,40) = 19.97; both *p* < 0.001) and 48 h (solvent: *F*(4,20) = 130.20; concentration: *F*(3,20) = 159.34; both *p* < 0.001). A significant solvent × concentration interaction was also evident (24 h: *F*(12,40) = 2.80, *p* = 0.007; 48 h: *F*(12,20) = 10.02, *p* < 0.001). Though post hoc comparisons found no statistically significant difference in viability between any HFHC treatment and the ethanol VC at any given concentration or time point (all adjusted *p* > 0.05). This suggests that none of the HFHC solvents reduced viability beyond the effect of ethanol itself. A one-way ANOVA on the vehicle-only data substantiates ethanol’s concentration-dependent toxicity (24 h: *F*(3,8) = 7.24, *p* = 0.012; 48 h: *F*(3,4) = 24.51, *p* = 0.003). In particular, one-sample *t*-tests against the 100% viability baseline (NC) showed that only the highest dose (2.5%) produced a statistically significant decrease in viability (~40.5% at 24 h, *p* = 0.001; and ~82.9% at 48 h, *p* = 0.026). While there was a modest viability decrease at 0.25% and 0.5% ethanol, they did not reach significance (unadjusted *p* ≈ 0.15 and 0.20, respectively), although a consistent downward trend with increasing dose emphasizes a subtle cumulative toxicity below the significance threshold.

Pearson’s correlation of the viability profiles strongly reinforced ethanol’s role as the primary toxic influence for DF-2000 and EcoSolv, with concentration–response curves almost superimposable on that of the VC (24 h: *r* ≈ 0.96; 48 h: *r* ≈ 0.99 for DF-2000; 48 h: *r* ≈ 0.93 for EcoSolv; *p* < 0.05 for each). In contrast, Calypsolv and Intense showed weaker similarity to the ethanol-only viability pattern, indicating potential HFHC-specific effects.

**Figure 1.**
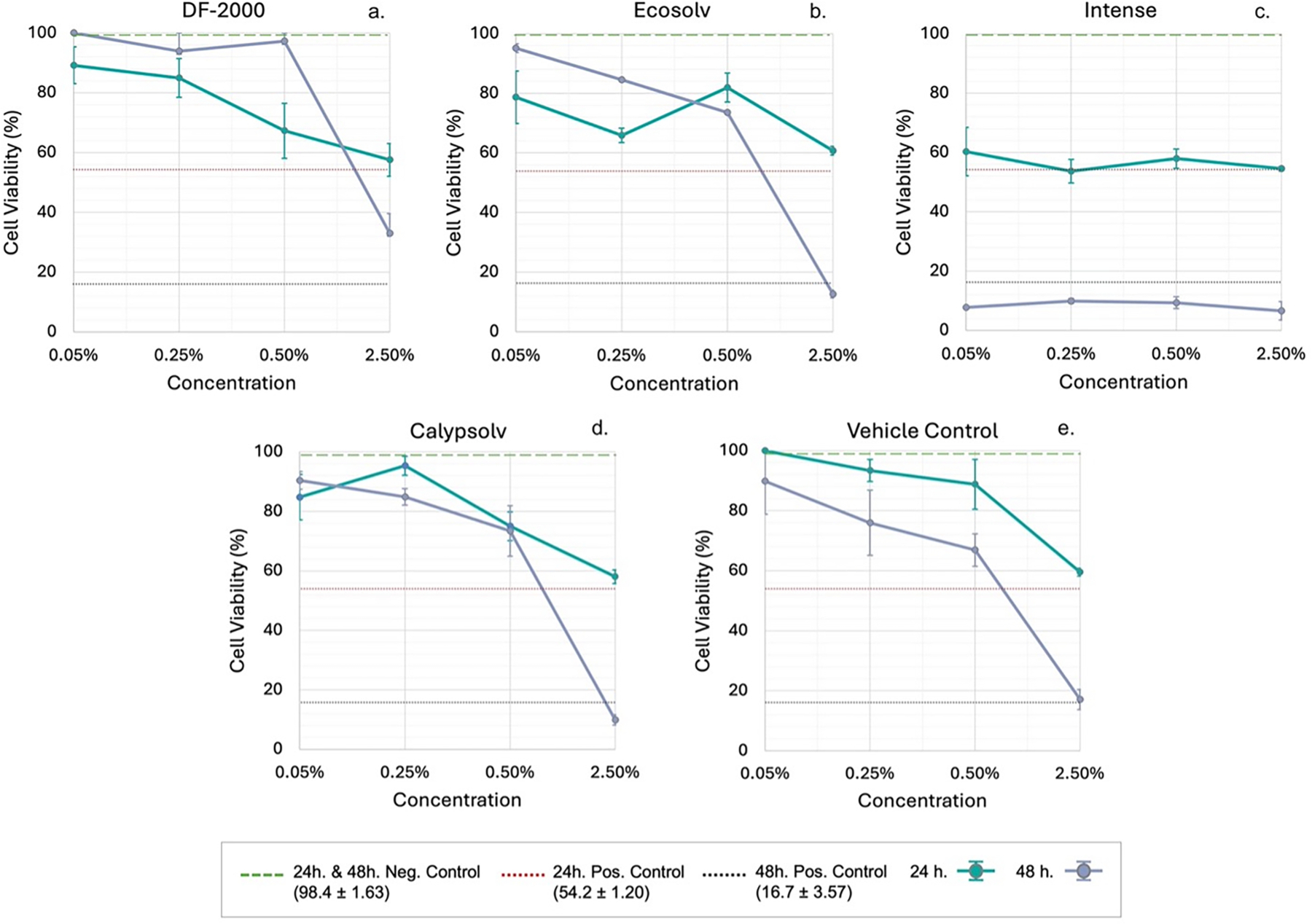
BEAS-2B cell viability (%) assessed by MTS assay following exposure to solvents DF-2000 (a), EcoSolv (b), Intense (c), Calypsolv (d), and vehicle control (e) at varying concentrations (% v/v) for 24 h (turquoise lines) and 48 h (purple lines). Negative control viability (untreated cells) indicated by green dotted line, positive control viability at 24 h by red dotted line, and at 48 h by gray dotted line with numerical mean values (±SE) provided in parentheses.

MTS results demonstrate that the ethanol cosolvent concentration, not the identity of the dissolved HFHC chemical, can act as the primary determinant of BEAS-2B cytotoxicity under the conditions tested. Figure 1 shows a comparative overview of cell viability across all treatments and concentrations at both 24 and 48 h, illustrating the dose-dependent declines and the overlapping responses of the HFHC-treated cells and the ethanol-only control at higher doses, with horizontal markers denoting the average 24 and 48 h NCs (both 100% viability), 24 h PCs, and 48 h PCs.

### 3.2. LDH assay: 24-hour exposure

**Figure 2.**
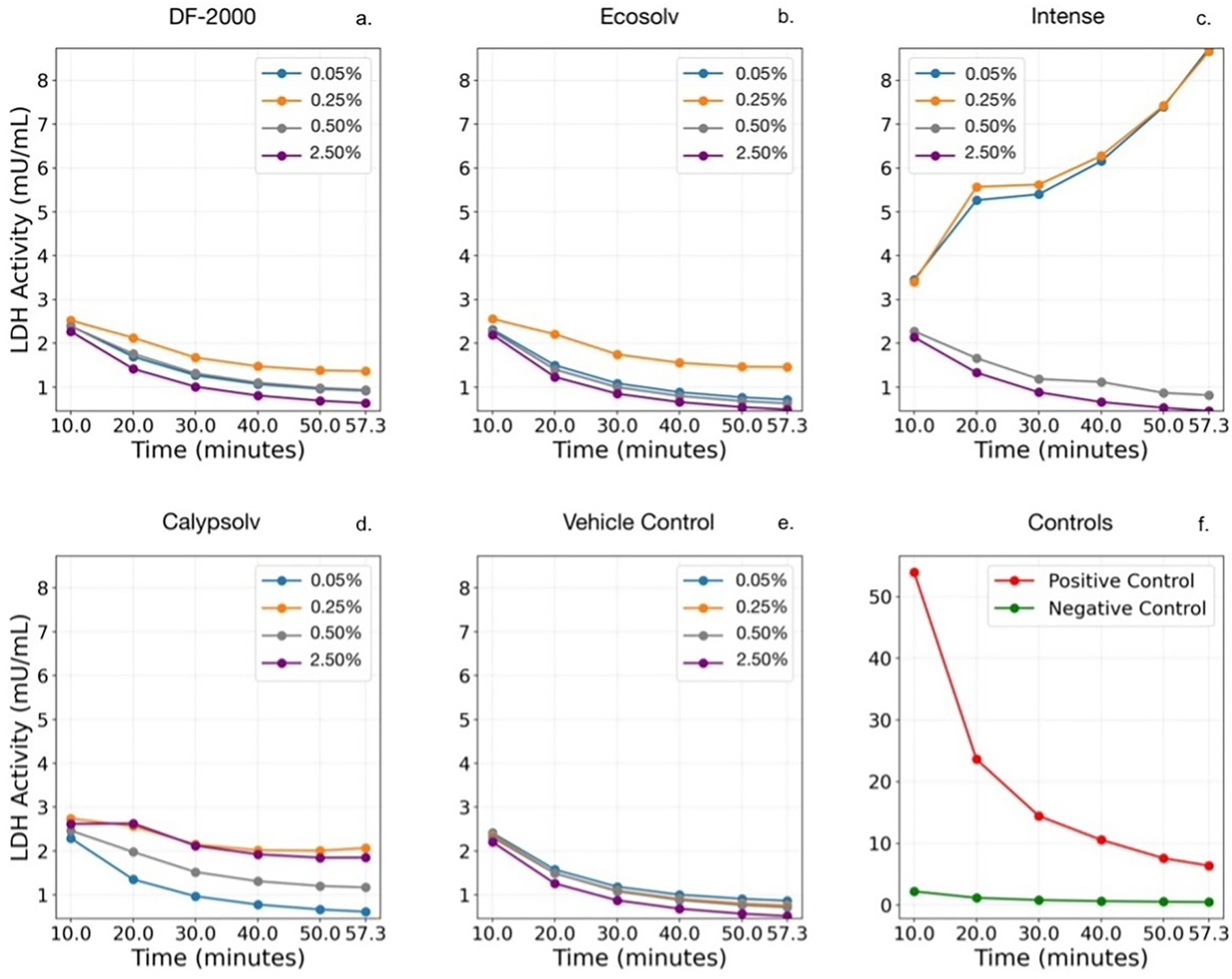
LDH activity (mU/mL) measured in real-time over 57.3 min following 24-hour BEAS-2B cell exposure to solvents DF-2000 (a), EcoSolv (b), Intense (c), Calypsolv (d), vehicle control (e), and positive and negative controls (f). Panels (a–e) depict LDH activity across varying concentrations (% v/v). Panel (f) shows LDH activity for positive (red) and negative (green) controls.

Following 24 h of exposure, the release of LDH into the culture medium was measured in real-time as an indicator of loss of membrane integrity; higher LDH readings indicate greater cytotoxic membrane damage. As shown in Figure 2, the majority of treatments (except Intense at 0.05% and 0.25%) exhibited LDH activity that peaked immediately after assay initiation and gradually declined over the following 57-minute measurement window. This pattern implies that the bulk of membrane rupture had occurred by 24 h and that fewer additional cells were lysing as the measurement period progressed, potentially due to enzyme consumption or stabilization of the remaining cells.

The ethanol VC produced only minimal LDH release, with an average of 1.20 ± 0.58 mU/mL across the 0.05–2.5% (v/v) VC and no dose-dependent increase (one-way ANOVA, *p* = 0.848). In comparison, specific HFHC-containing formulations induced far greater LDH leakage that was unmistakably attributable to the formulation rather than the ethanol vehicle. Particularly, Intense at 0.05% and 0.25% provoked an approximately fivefold greater LDH release compared to ethanol alone. Post hoc comparisons confirmed that Intense’s LDH elevations at these low concentrations were significantly greater than those observed with the VC (Bonferroni-adjusted *p* = 0.0027 at 0.05%; *p* = 0.0019 at 0.25%) as well as versus the other HFHCs, EcoSolv (*p* = 0.0024 and 0.0045 at 0.05% and 0.25%, respectively) and DF-2000 (*p* = 0.0030 and 0.0041). The HFHC solvent Calypsolv elicited a reserved LDH increase: its peak release (~2.26 ± 0.32 mU/mL at 0.25%) was roughly double the VC level but still remained far below Intense. No appreciable LDH elevation was observed with Intense at higher concentrations (0.5% and 2.5%) or with any concentration of EcoSolv or DF-2000, showing that ethanol had a negligible cytotoxic impact. The enhanced LDH release in specific treatments (especially Intense and, to a lesser extent, Calypsolv) is distinguishable from the minimal effect of ethanol as a cosolvent. Overall, the degree of membrane damage corresponded to the specific chemical solution rather than the ethanol vehicle.

### 3.3. IL-8 cytokine test: 24-hour exposure

After 24 h of exposure, IL-8 cytokines, pro-inflammatory markers of cellular stress and immune activation, were measured in the cell culture supernatants of the exposed BEAS-2B cells. Any heightened readings of IL-8 provide insights into cytotoxic reactions resulting from interaction with a pro-inflammatory stimulus (Bickel 1993; Vilotić et al. 2022). A two-way ANOVA confirmed that IL-8 release was significantly affected by ethanol concentration (*F*(3,35) = 121.49, *p* < 0.001). In contrast, solvent identity (ethanol vs. any of the four HFHC solvents) had no significant overall effect on IL-8 levels (*F*(4,35) = 0.59, *p* = 0.562). A considerable concentration × solvent interaction was observed (*F*(12,35) = 7.41, *p* < 0.001), but post hoc Tukey’s HSD tests found no significant differences between IL-8 responses in any HFHC treatment and the corresponding ethanol VC at the same concentration (all adjusted *p* > 0.05). For example, at the highest ethanol level (2.5%), IL-8 concentrations in the ethanol VC and all HFHC solvent groups were virtually identical (~6.9 pg/mL), indicating that none of the HFHC treatments induced IL-8 release beyond the level caused by ethanol alone, reason to believe that ethanol concentration was the dominant factor influencing IL-8 secretion, rather than the HFHC solvent type.

Within the ethanol VC group, IL-8 secretion increased in a clear dose-dependent manner from 0.05% to 2.5% (one-way ANOVA: *F*(3,7) = 8.33, *p* = 0.0104). Further, at the lowest concentration (0.05%), IL-8 reached 5.59 ± 0.76 pg/mL, ~87% of the overall mean IL-8 level observed across all treatment conditions (6.40 pg/mL). Therefore, even minimal ethanol exposure produced a high baseline IL-8 response, and supplemental increases in ethanol concentrations yield only modest additional IL-8 release (e.g. IL-8 at 2.5% ethanol was ~7.0 pg/mL, just 25% above the level at 0.05%).

**Figure 3.**
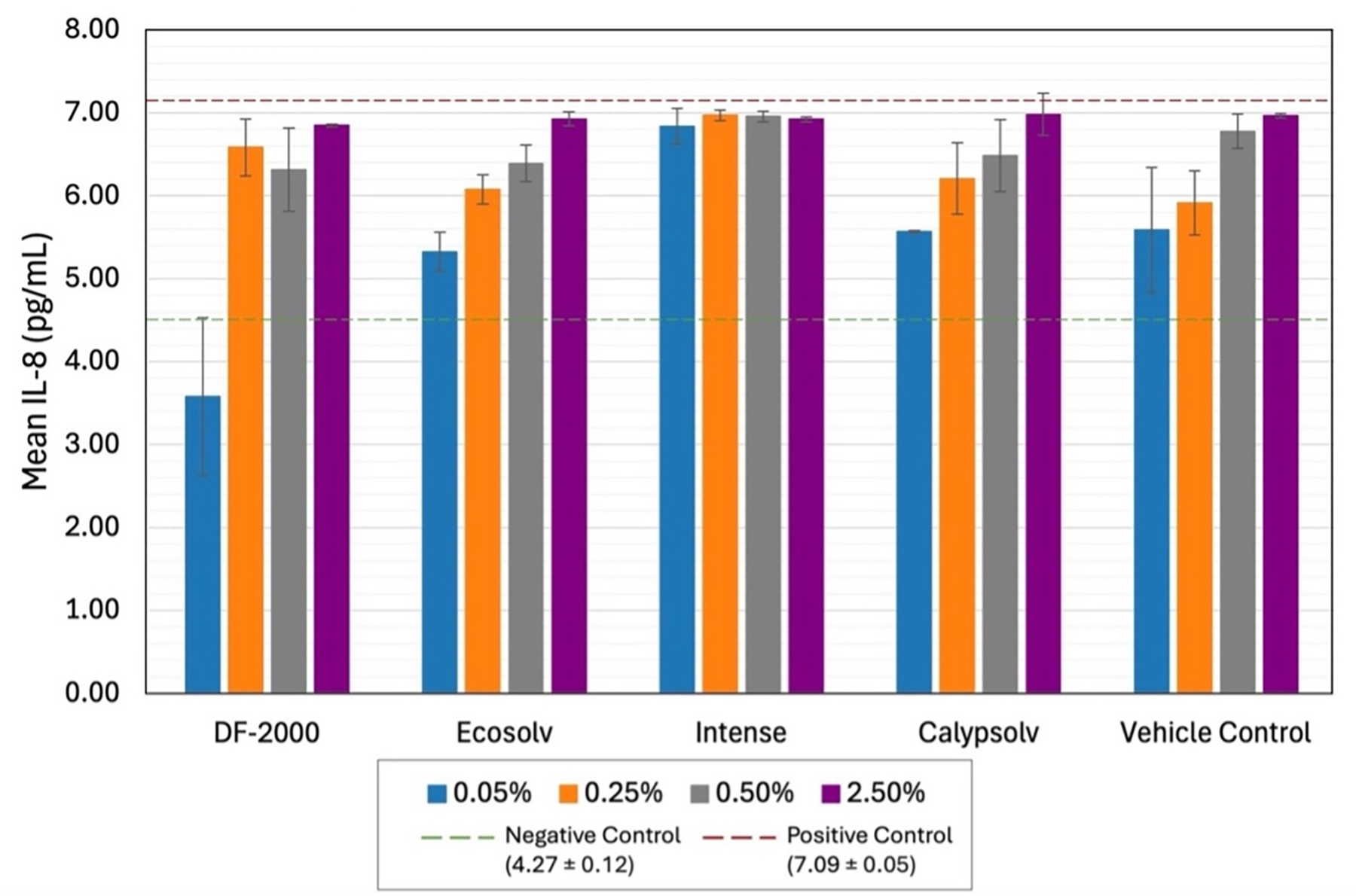
Mean interleukin-8 (IL-8) cytokine concentrations (pg/mL) in BEAS-2B cells following 24-hour exposure to solvents (DF-2000, EcoSolv, Intense, and Calypsolv) and vehicle control at concentrations of 0.05%, 0.25%, 0.5%, and 2.5% (v/v). Dashed lines indicate mean IL-8 levels for negative (green) and positive (red) controls, with numerical mean values (±SE) provided in parentheses.

Pearson’s correlation analysis validated that IL-8 elevations were primarily driven by ethanol, with IL-8 responses for both Calypsolv and EcoSolv strongly correlated with those of the VC across concentrations (*r* = 0.93, *p* = 0.066 for each), mirroring the dose–response patterns observed with ethanol alone. In contrast, IL-8 levels for DF-2000 (*r* = 0.73, *p* = 0.265) and Intense (*r* = 0.49, *p* = 0.508) had lower, non-significant correlations with the ethanol VC, potentially reflecting unique inflammatory mechanisms. However, overall, the findings suggest that ethanol itself was the principal contributor to IL-8 secretion, with the HFHC solvents contributing little to no additional IL-8 induction beyond the effect of ethanol. These trends are illustrated in Figure 3, which depicts IL-8 levels increasing similarly with ethanol concentration for the VCs and each HFHC solvent, portraying nearly overlapping dose–response curves.

## 4. Discussion

*In vitro* toxicological screening of hydrophobic environmental contaminants requires vehicles that effectively solubilize test compounds without introducing confounding cellular responses. In this study, we characterized ethanol’s safety window in BEAS-2B human bronchial epithelial cells by evaluating four emerging HFHC dry-cleaning chemicals (Calypsolv, EcoSolv, DF-2000, and Intense) to determine potential vehicle-specific effects. This was achieved by quantifying metabolic activity (MTS assay), membrane integrity (LDH release), and inflammatory signaling (IL-8 secretion) across varying matched concentrations (0.05–2.5% v/v).

### 4.1. Cell viability

In the absence of HFHC chemicals, ethanol VC showed concentration-dependent cytotoxicity. Short-term (24-hour) exposure to up to 0.5% (v/v) caused minimal loss of viability (88.76 ± 8.30%), whereas exposure to 2.5% caused a pronounced drop of ~40%. Statistically, 0.5% did not produce a significant deviation from the untreated control at 24 h, indicating a 24-hour NOAEL at this dose in BEAS-2B. However, at 2.5%, viability fell to ~60% of the NC, a substantial cytotoxic effect, pin-pointing the 24-hour LOAEL ≤2.5% under our conditions. Prolonged exposure at 48 h greatly sensitized the cells, as even 0.5% ethanol, which had been benign at 24 h, became overtly cytotoxic by 48 h (down to ~67%). At the highest dose (2.5%), increased duration reflected near-total cell loss (~17%), comparable to the toxic PC (~16%). Thus, when extending exposure to 48 h, the NOAEL is lowered to 0.25% ethanol, and the LOAEL to 0.5% for BEAS-2B cells. These shifts demonstrate that both dose and exposure time must be carefully considered when assessing viability.

None of the four HFHC solvents significantly exacerbated or protected against ethanol’s inherent cytotoxicity in terms of cell viability. Whether cells were exposed to ethanol alone or ethanol carrying a HFHC chemical, the viability loss was equivalent to each ethanol percentage, an indicator that ethanol’s cytotoxic action wholly accounts for the observed loss of viability, as the HFHCs themselves did not measurably contribute additional cytotoxicity in these conditions. One exception was the HFHC, Intense, which appeared to cause an out-of-pattern drop in viability at the lowest doses in the 24-hour test where at 0.05% (v/v) viability was reduced to ~60% of the control, vs. ~80–100% observed with the other three HFHCs and VC at the same dose. This did not remain statistically significant after correcting for multiple comparisons, but it suggests a compound-specific cytotoxic effect that manifests even at ostensibly nontoxic ethanol levels. By 0.25–0.5%, Intense-treated wells continued to show depressed viability (~54–58% viable) relative to other treatments, whereas other HFHCs tracked closely with the VC.

Despite Intense’s outlier behavior, ethanol concentration still explained the majority of the cytotoxic effect. By the highest dose (2.5%, 24 h), all conditions (including Intense) converged to near-complete cytotoxicity (~55–60% viability across groups), indicating that ethanol at 2.5% overwhelms cells regardless of any additive HFHC toxicity. Likewise, after 48 h, Intense’s extreme cytotoxicity persisted, but other HFHC exposures also led to severe viability losses at 2.5% (e.g. only ~10–33% viable with Calypsolv, EcoSolv, and DF-2000). These findings reinforce that ethanol’s own toxicity was the dominant factor, while HFHC-specific effects were secondary. Our data suggest that for BEAS-2B, ~0.5% (v/v) is the upper limit for a 24-hour exposure without appreciable cytotoxicity, dropping to ~0.25% for 48 h, thresholds more conservative than some reported for other non-tumorigenic cell types.

### 4.2. Membrane integrity

The LDH assay provided insight into membrane damage and revealed distinct patterns for specific HFHCs rather than ethanol. In the VC wells, LDH release remained at baseline levels at low concentrations (≤0.5%), and even at 2.5% ethanol, the increase in LDH was only moderate. In contrast, Intense caused a dramatic LDH surge at the lowest concentrations, with release roughly fivefold higher than in the corresponding VCs. We can conclude that Intense uniquely compromises cell membranes even at doses that, for ethanol alone, are non-disruptive. The peak LDH activity with Intense (~6.1 mU/mL at 0.25%) shadowed that of ethanol (~1.2 mU/mL) at the same concentration, a statistically significant elevation. Calypsolv elicited a modest membrane-disruptive effect, with its highest LDH release (~2.3 mU/mL at 0.25%) being double the VC, while the other two HFHC solvents (DF-2000 and EcoSolv) showed no appreciable LDH elevation above the baseline.

Membrane integrity was largely preserved with ethanol; notably, even at the highest ethanol dose tested (2.5%), LDH release remained minimal and statistically indistinguishable from baseline (*p* = 0.848). Therefore, the extent of LDH leakage observed at higher doses depended primarily on the specific HFHC formulation rather than the ethanol carrier. The fact that Intense and (to a lesser extent) Calypsolv caused greater LDH release than ethanol suggests that certain chemical constituents in these mixtures can interact with or disrupt cell membranes more aggressively than ethanol. Intense is an aliphatic hydrocarbon solvent, but its exact composition (proprietary blend) may include fractions that are membrane-perturbing; this could explain the acute membrane lysis observed. Correlatively, Intense’s pronounced LDH release at 0.05–0.25% coincided with significant viability loss in those groups, implicating membrane rupture as a mechanism for its cytotoxicity. For instance, cells exposed to 0.05% Intense showed ~5× higher LDH and ~40% lower viability than the VC, a parallel suggesting that Intense’s early cell killing was driven by membrane damage.

Most treatments peaked early during the assay measurement and then declined, signifying that the bulk of susceptible cells had already ruptured by 24 h. This saturation of effect at high doses is consistent with the notion that there is a finite population of cells to kill; once a critical fraction of membranes is compromised, additional solvent cannot further increase LDH or decrease viability. While ethanol is generally a benign vehicle at low percent volumes, one must also consider the possibility that the dissolved chemical might have its own membrane-active properties, necessitating careful vehicle comparisons. The membrane integrity endpoint proved more sensitive to formulation-specific toxicity than did the viability endpoint; for example, Intense’s unique effects were more readily apparent in LDH release than in overall MTS viability.

### 4.3. Inflammatory response

Ethanol’s impact on cellular function was most strikingly revealed in the IL-8 cytokine assay, with release driven almost entirely by ethanol concentration, and no significant contribution from the identity of the HFHCs. Statistical analyses confirmed that while increasing ethanol caused highly significant changes in IL-8 levels (*p* < 0.001), there were no differences between any HFHC-treated group and the corresponding ethanol-only VC at the same dose. The pattern of IL-8 release across ethanol doses was non-linear. Even the lowest ethanol concentration tested (0.05% v/v) elicited a substantial IL-8 response, ~5.6 pg/mL, ~87% of the mean IL-8 level observed across all treatments. Increasing concentrations did not significantly alter levels, as IL-8 essentially plateaued by the lowest dose, with only small incremental gains at higher concentrations. This response suggests that BEAS-2B cells mount an acute inflammatory signaling response to ethanol at very low exposure, saturating quickly.

One explanation is that even minimal ethanol can activate stress pathways (e.g. oxidative stress or membrane perturbation leading to NF-κB activation), triggering the release of IL-8. Once those pathways are engaged, additional ethanol primarily causes cytotoxicity rather than further cytokine upregulation. Data show that beyond 0.5–2.5%, many cells were dying (as evidenced by MTS and LDH assays), limiting their capacity to secrete cytokines. Thus, we can conclude that sub-toxic ethanol levels induce IL-8, whereas overtly toxic levels kill cells, an inverse relationship.

This interpretation aligns with reports that ethanol can disrupt cellular signaling and cytokine balance at intermediate concentrations (around 0.5–1.0% in other models) even when viability appears unaffected.

IL-8 findings identify that even ‘vehicle-level’ ethanol (≤0.5%) is not biologically inert in BEAS-2B cells; it can elevate pro-inflammatory IL-8. This carries major implications, as researchers measuring inflammatory mediators could be misled if an ethanol VC is omitted, since a test compound dissolved in, say, 0.5% ethanol might appear to induce cytokines when, in reality, ethanol alone is capable of doing so. Notably, two of the HFHC solvents (Calypsolv and EcoSolv) showed IL-8 dose–response curves strongly resembling ethanol’s pattern (*r* ≈ 0.93), although correlations narrowly missed statistical significance (*p* = 0.066), indicating a highly similar, though not conclusively identical, inflammatory response. Mechanistically, this indicates ethanol can act as a pro-inflammatory stimulus in epithelial cells at low doses, an effect that does not scale proportionally with higher doses due to the onset of cell death.

### 4.4. Implications for ethanol vehicle in BEAS-2B models

Findings portray that small percentages of ethanol can have outsized biological effects in certain sensitive cell models. BEAS-2B cells showed both cytotoxic and inflammatory responses to ethanol at concentrations that are below, within, or just above the range commonly used to solubilize hydrophobic compounds (0.1–1% v/v). From the conducted BEAS-2B model, we can recommend that ethanol be limited to ≤0.5% for 24-hour exposures and ≤0.25% for 48-hour exposures, when utilizing viability and membrane integrity assays, and ≤0.05% for inflammatory cytokine tests. Exceeding these thresholds jeopardizes confounding the experiment with vehicle-induced cytotoxicity or spurious cytokine release. Although some prior studies have tolerated higher ethanol in more robust cell lines, our data caution that BEAS-2B is more vulnerable, likely reflecting their physiological, untransformed state. Thus, guidelines for cosolvent use should not be one-size-fits-all; the NOAEL should be established for each cell type of interest.

At the same time, a few considerations limit the broad applicability of these recommendations. While BEAS-2B cells are a widely accepted human bronchial epithelial model, they may not fully replicate the physiological complexity of primary human tissue or *in vivo* systems. Additionally, the concentration thresholds identified here are specific to the three endpoints evaluated (viability, membrane integrity, and IL-8 cytokine secretion) and may not necessarily extend to assays targeting other cellular responses or mechanistic pathways. Lastly, only four proprietary HFHC solvents were evaluated; additional testing may reveal different vehicle–mechanism interactions with different pollutants of other chemical classes.

Even with such limitations, the current findings strongly reinforce the importance of including VCs. Any study employing ethanol as a carrier must include parallel wells with ethanol alone at the same concentration used in treatment groups. In this experiment, the matched VCs were indispensable for isolating ethanol’s effects, revealing IL-8 elevation was likely entirely a vehicle effect, and that a HFHC like Intense had unique toxicity only detectable by comparison to ethanol-only baseline. VCs ensure that any observed biological effect can be correctly attributed to the test compound rather than the solvent. If a particular solvent (like ethanol) is found to exert appreciable effects of its own, researchers might consider alternative solubilization strategies (e.g. DMSO or PEG, reducing percentage by increasing stock concentration, or utilizing encapsulation or emulsification techniques).

Intense and Calypsolv imparted extra membrane stress beyond ethanol, suggesting that when testing novel or proprietary chemical mixtures, one should evaluate the mixture’s intrinsic toxicity separately from the solvent vehicle. In our case, we disentangled these by comparing each HFHC + ethanol treatment to ethanol alone; a similar approach is advisable whenever the dissolving vehicle is suspected to interact with cells. Aside from Intense’s low-dose outlier effect, the other HFHC solvents did not significantly deviate from ethanol controls in viability or IL-8 endpoints. Intense serves as a reminder that some vehicle-analyte combinations can have synergistic or unexpected effects on cells (perhaps due to improved delivery of the analyte or additive toxicity), meriting case-by-case scrutiny.

The demonstration of a plateau in IL-8 response and LDH at high ethanol doses carries mechanistic implications, both suggesting a limit to how much stress response a cell population can mount once a critical fraction is compromised. Increasing ethanol (or any vehicle carrier) concentrations in a cell assay will not necessarily result in a proportional increase in a functional endpoint. For example, when examining inflammatory signaling, using 2.0% ethanol may mask a compound-induced cytokine response because ethanol alone triggers near-maximal IL-8 and subsequently kills cells. Thus, adhering to sub-cytotoxic solvent levels is not only about avoiding false-positive toxicity, but also about preserving the assay’s sensitivity to detect the true effects of the test compounds.

## 5. Conclusions

In conclusion, this study demonstrates that ethanol, at standardly used concentrations, can serve as a driver of confounding cytotoxicity and IL-8 inflammatory signaling in BEAS-2B cells when implemented as a cosolvent. The addition of four HFHC dry-cleaning solvents, except for one (Intense), did not meaningfully affect viability, membrane integrity, or IL-8 release beyond the effects of ethanol alone. Based on the MTS and LDH assays, we identified practical solvent noninterference thresholds, recommending that future *in vitro* studies using BEAS-2B cells limit ethanol to approximately 0.5% (v/v) for 24-hour exposures and up to 0.25% for 48-hour exposures. Separately, IL-8 cytokine results revealed that extremely low ethanol levels can induce a baseline pro-inflammatory response, highlighting that ethanol can confound sensitive endpoints like cytokine release even when overt cytotoxicity is absent. Therefore, we recommend limiting ethanol to ≤0.05% when running inflammatory cytokine tests. Sticking to these approximate thresholds will help ensure that any observed toxicological effects are attributable to the test compound, assisting researchers in selecting suitable dissolution strategies and ethanol cosolvent limits while enhancing the accuracy of toxicity assessments for environmental pollutants.

## Figures and Tables

**Table 1.** Summary table of changes in cell viability: 24 h of exposure.

Exposures	–	0.05%	0.25%	0.50%	2.50%
Positive control	54.23 ± 1.20				
Negative control	98.15 ± 1.85				
Calypsolv		84.79 ± 7.65	95.33 ± 3.20	75.00 ± 4.81	58.08 ± 2.30
DF-2000		89.20 ± 6.13	84.97 ± 6.53	67.30 ± 9.21	57.58 ± 5.49
EcoSolv		78.66 ± 8.78	65.85 ± 2.43	81.94 ± 4.87	60.73 ± 1.49
Intense		60.29 ± 8.15	53.72 ± 3.99	57.95 ± 3.26	54.55 ± 0.83
Vehicle control		100 ± 0.0	93.37 ± 3.67	88.76 ± 8.30	59.53 ± 1.30

The Beas-2B cells were treated with 0.05%, 0.25%, 0.5%, or 2.5% of HFHC solvents, each with a 1:1 ratio of ethanol for dissolution. The data in Table 1 are expressed as percentages of viable cells, normalized to the negative control samples. The results are expressed as mean ± standard error, n = 3.

**Table 2.** Summary table of changes in cell viability: 48 h of exposure.

Exposures	–	0.05%	0.25%	0.50%	2.50%
Positive control	16.56 ± 3.57				
Negative control	98.58 ± 1.42				
Calypsolv		90.40 ± 2.92	84.86 ± 2.75	73.41 ± 8.50	9.88 ± 1.68
DF-2000		100 ± 0.0	93.89 ± 6.11	97.24 ± 2.76	32.99 ± 6.61
EcoSolv		95.14 ± 1.44	84.50 ± 0.35	73.55 ± 0.34	12.63 ± 1.31
Intense		7.73 ± 0.48	9.95 ± 0.79	9.35 ± 2.01	6.61 ± 3.13
Vehicle control		89.81 ± 11.02	75.94 ± 10.85	66.91 ± 5.39	17.10 ± 3.36

The BEAS-2B cells were treated with 0.05%, 0.25%, 0.5%, or 2.5% of HFHC solvents, each with a 1:1 ratio of ethanol for dissolution. The data in Table 2 are expressed as percentages of viable cells normalized to the negative control samples. The results are expressed as mean ± standard error, *n* = 2.

## Data Availability

An overview of the data generated and analyzed in this study is provided within the published article. Detailed raw data and complete analysis are available from the corresponding author upon request.
